# Supplementary material for: Borealin–nucleosome interaction secures chromosome association of the chromosomal passenger complex
Source: J Cell Biol. 2019 Sep 30;218(12):3912–25. doi: 10.1083/jcb.201905040 (PMC6891087; doi:10.1083/jcb.201905040)
Supplement: Supplemental Materials (PDF) [file JCB_201905040_sm.pdf]

Supplemental material

Abad et al., https://doi.org/10.1083/jcb.201905040

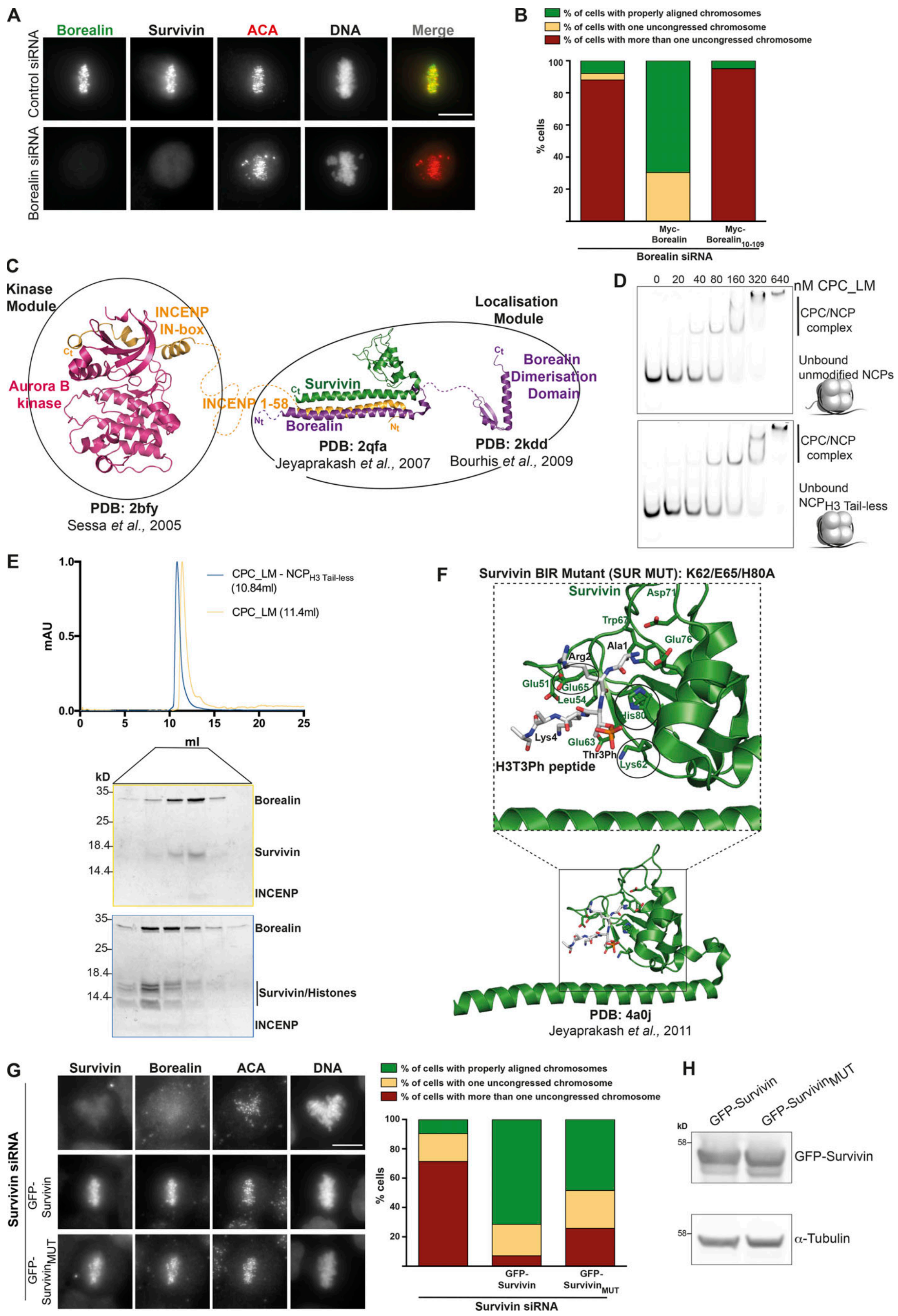

Figure S1. **Structural organization of the CPC.** **(A)** Treatment of siRNA oligonucleotides targeting Borealin 3′ UTR resulted in no Borealin signal from early prophase to anaphase. Immunofluorescence staining of Borealin, Survivin, and ACA in HeLa cells. Hoechst was used for DNA staining. Images were taken 36 h after transfection. Scale bar, 10 µm. **(B)** Analysis of cells showing uncongressed chromosomes in the siRNA-rescue assay for Myc-Borealin$_{10–109}$ fragment shown in Fig. 1 B. A total of 25, 23, and 20 cells were counted for Borealin siRNA, Myc-Borealin, and Myc-Borealin$_{10–109}$ rescue conditions, respectively. **(C)** The CPC can be divided into a localization module (composed of Borealin, Survivin, and INCENP 1–58) and a kinase module (composed of Aurora B and INCENP IN-box) connected by a central helical coiled coil of INCENP. Cartoon representations of the structures were generated using Pymol v2.0.6. **(D)** Native PAGE analysis of EMSA assays performed with increasing concentrations of CPC_LM with either unmodified or H3-tailless (NCP$_{H3\ Tailless}$) NCPs. **(E)** Size exclusion chromatogram of recombinant CPC_LM (yellow) and CPC_LM in complex with NCP$_{H3\ Tailless}$ (blue) using a Superdex 200 increase 10/300 GL column (top). Coomassie-stained SDS-PAGE analysis of samples resolved in the Superdex 200 column (bottom). mAU, milli-Absorbance Unit. **(F)** Close-up view of the histone H3 peptide phosphorylated at Thr3 (gray) bound to Survivin (green; PDB accession no. 4A0J; Jeyaprakash et al., 2011). Survivin residues involved in H3 tail binding are shown in stick representation. Amino acid residues mutated in the BIR domain of Survivin (SUR MUT: K62/E65/H80A) to abolish H3-tail binding are highlighted in circles. **(G)** Representative fluorescence images of a Survivin siRNA rescue assay for GFP-Survivin and GFP-Survivin BIR mutant (K62/E65/H80A; GFP-Survivin$_{MUT}$). Immunofluorescent staining of Survivin, Borealin, and ACA in HeLa cells cotransfected with 50 nM siRNA duplexes targeting the 3′ UTR region of Survivin and 300 ng of GFP-Survivin constructs. Hoechst was used for DNA staining. Scale bar 10 µm (left). Quantification of uncongressed chromosomes observed for the siRNA-rescue assay of GFP-Survivin and GFP-Survivin$_{MUT}$ shown in the left panel (right). A minimum of 25 cells were counted. **(H)** Analysis of GFP-Survivin and GFP-Survivin$_{MUT}$ expression levels by Western blot.

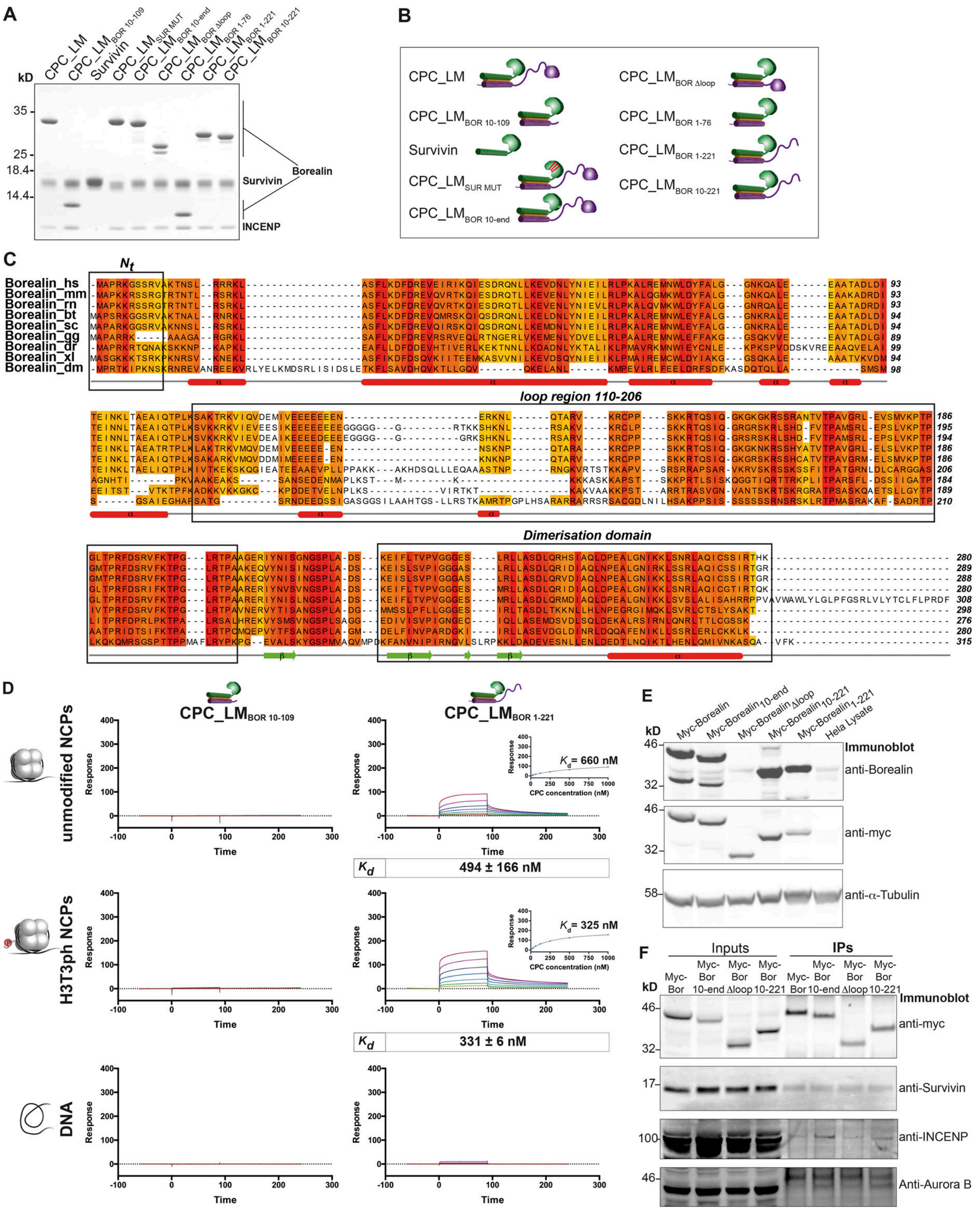

Figure S2. **Multiple regions of Borealin contribute to nucleosome binding. (A)** Representative Coomassie-stained SDS-PAGE analysis of purified recombinant CPC complexes used in the EMSA and SPR assays. **(B)** Schematic cartoon diagrams for various CPC variants used in this study. **(C)** Amino acid conservation of Borealin (conservation score is mapped from red [highly conserved] to yellow [poorly conserved]). The alignment includes Borealin orthologues from *Homo sapiens* (hs), *Mus musculus* (mm), *Rattus norvegicus* (rn), *Bos taurus* (bt), *Sus scrofa* (ss), *Gallus gallus* (gg), *Danio rerio* (dr), *X. laevis* (xl), and *Drosophila melanogaster* (dm). Predicted secondary structure elements are depicted below the sequence alignment. The black box highlights regions that are critical for nucleosome binding. Multiple sequence alignment was performed with Clustal Omega (EMBL-EBI) and edited with Jalview 2.10.5 (Waterhouse et al., 2009). **(D)** Representative SPR sensorgrams of the interaction between different CPC_LM complexes (CPC_LM$_{BOR\ 10-109}$ and CPC_LM$_{BOR\ 1-221}$) and unmodified (top) or H3T3ph (middle) NCPs or DNA (bottom). Mean values ($n \geq 3$, ±SEM) determined for the equilibrium K_d are shown in boxes underneath the sensorgrams. **(E and F)** Representative immunoblots showing the expression levels (E) and the complex formation with other CPC components (F) of the different Myc-Borealin constructs used in Fig. 2 C. Note that the Borealin antibody used did not recognize the Borealin$_{\Delta loop}$ protein, so the anti-myc antibody was used to recognize this fragment.

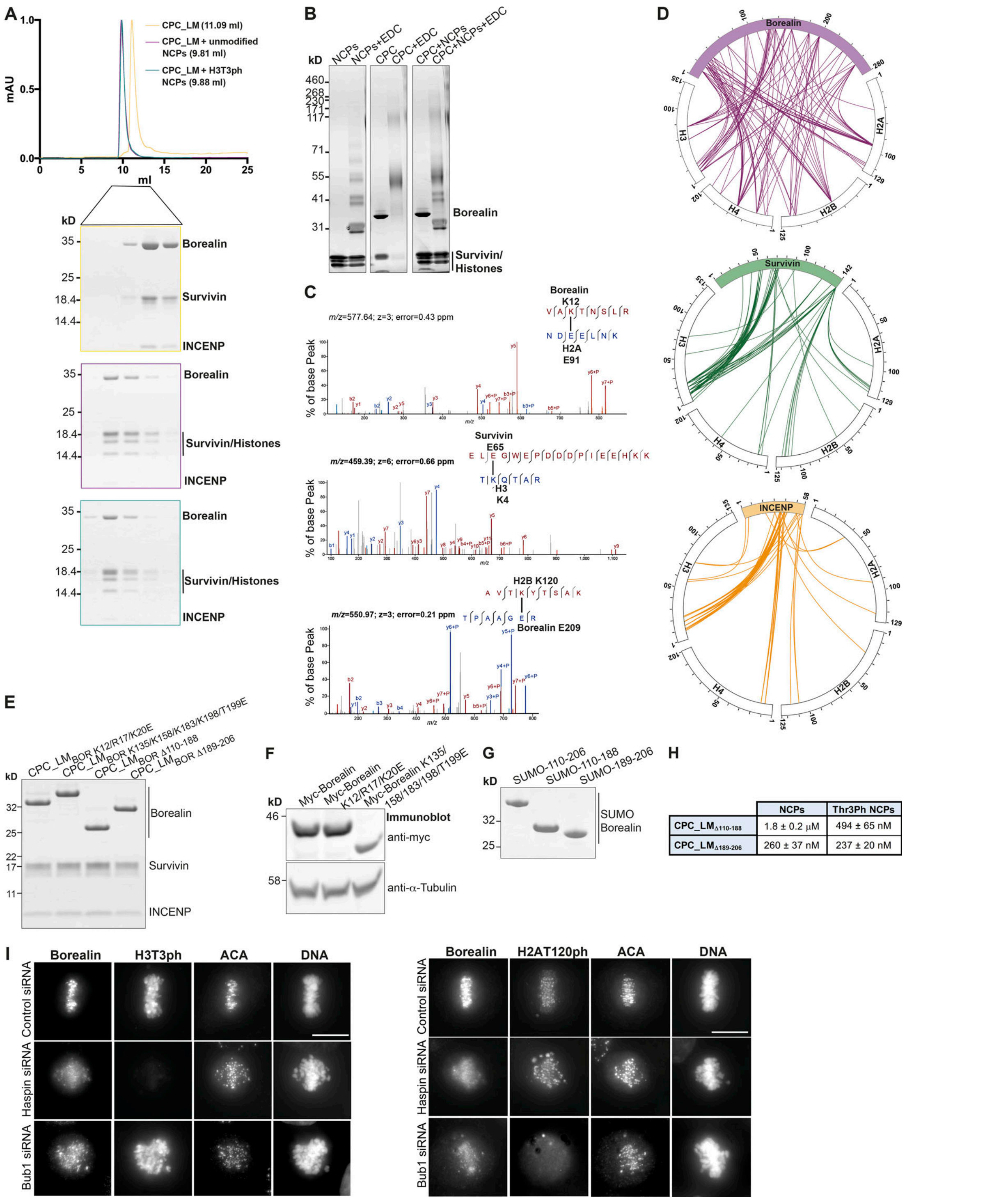

Figure S3. **Biophysical and cellular characterization of Borealin mutants in facilitating nucleosome binding and chromosome association of CPC.** **(A)** Size exclusion chromatogram of recombinant CPC_LM (yellow) and CPC_LM in complex with unmodified (magenta) or H3T3ph (blue) NCPs obtained using a Superdex 200 increase 10/300 GL column (top). Coomassie-stained SDS-PAGE analysis of samples resolved in the Superdex 200 column (bottom). **(B)** Representative SDS-PAGE of 8 µg of CPC or CPC-NCP complex cross-linked with EDC cross-linker. **(C)** High-resolution fragmentation spectra of a few representative cross-linked peptides, displayed using XiSpec. **(D)** Cross-link mapping of interactions between the CPC subunits (Borealin, purple; Survivin, green; INCENP, yellow) and histones from H3T3ph NCPs. Intermolecular contacts involving Borealin, Survivin, INCENP, and histones are shown as purple, green, or yellow lines. **(E)** Coomassie-stained SDS-PAGE analysis of purified recombinant CPC complexes used in the SPR assays in Fig. 4 A. **(F)** Analysis of the expression levels of the Myc-Borealin constructs used in Fig. 4 (B and C). **(G)** Coomassie-stained SDS-PAGE analysis of purified recombinant CPC complexes used in the SPR assays in Fig. 4 E. **(H)** Table showing the mean values ($n \geq 3$, ±SEM) determined for the equilibrium K_d for SPR analysis of CPC_LM constructs lacking the N-terminal ($CPC\_LM_{\Delta 110-188}$) and C-terminal ($CPC\_LM_{\Delta 189-206}$) part of the loop. **(I)** Immunofluorescence analysis of Borealin, H3T3ph, H2AT120ph, and ACA in HeLa cells transfected with control siRNA duplexes and siRNA duplexes targeting the Haspin or the Bub1 transcript for 36 h. Hoechst staining was used to visualize DNA. Bars, 10 µm.

References

Jeyaprakash, A.A., C. Basquin, U. Jayachandran, and E. Conti. 2011. Structural basis for the recognition of phosphorylated histone h3 by the survivin subunit of the chromosomal passenger complex. *Structure*. 19:1625–1634. https://doi.org/10.1016/j.str.2011.09.002

Waterhouse, A.M., J.B. Procter, D.M. Martin, M. Clamp, and G.J. Barton. 2009. Jalview Version 2--a multiple sequence alignment editor and analysis workbench. *Bioinformatics*. 25:1189–1191. https://doi.org/10.1093/bioinformatics/btp033
